# Bedford and Its Medical Institutions

**Published:** 1893-02-11

**Authors:** 


### HOSPITAL OPINION.

[Contributions critical, suggestive, controversial, and technical are invited for this column, which is open to everyone who has anything to say that is of practical value and interest.]

### BEDFORD AND ITS MEDICAL INSTITUTIONS.

"A Bedfordian" writes: Permit me, as one who takes an interest in Bedford and in its "Hospital-trained Nurses' Institute," to make a few comments upon the article which appeared in your issue of the 4th inst., entitled "Bedford and its Medical Institutions." Your Special Commissioner says that Bedford has "two provident dispensaries, each of which has attached to it a nurses' institute, and neither of these institutions has its nurses trained at the Royal Infirmary." Again, when speaking particularly of the Bedford Hospital-trained Nurses' Institute, he says, "Miss Rawson is the Honorary Lady Superintendent. . . . There can be no doubt that it would be most desirable to attach the Nurses' Institute to the Infirmary, and to make arrangements that its board should train the nurses there. This would give a much better guarantee of efficiency to those who require the services of a private nurse." There is much in the report which is generally accurate, and needs neither correction nor comment. I proceed, with your permission, to deal with the two extracts set out above. First, let me say that there is no Royal Infirmary here. The General Infirmary that your Special Commissioner speaks of is the only infirmary here, and it quite deserves all the favourable notice that he has given it. Its medical staff and its nursing staff under Miss Wemys are all that can be desired, and need no praise from me. The Lady Superintendent of the Hospital-trained Nurses' Institute is not Miss but Mrs. Rawson, the widow of a Chaplain on the Bengal Establishment, who is herself a trained nurse, trained at the Middlesex Hospital, and who for some months was the Sister-in-Charge of one of the galleries of the Brompton Hospital for Consumption. She has for the last four years, that is ever since the starting of this institute, most generously given her valuable services without payment to the institute; herself taking up the nursing of any case when the staff of nurses at her disposal have all been otherwise fully engaged. The committee, having the fullest confidence in Mrs. Rawson, have wisely left the selection of the nurses entirely in her hands, satisfied that her ability, judgment, and experiences are such as to guarantee that the nurses she may engage are efficient. Seeing that all these nurses are as the name of the institute states "hospital-trained nurses," it is difficult to conceive how the training of them by the "board" of the infirmary could increase their efficiency or guarantee it better than the course now adopted does. Bedford has been well described by its much respected representative in Parliament, Mr. Whitbread, as "the Metropolis of Education in England." Even now close upon, if not quite, 5,000 young people attend its schools, and the number grows year by year. In the interests as well of Mrs. Rawson's most excellent institution as of the schools and of the town itself, it is most desirable that no hinderance to the growth of the town should be created by a belief in the minds of the public that there is any lack here of well-trained and efficient nurses for the sick. No county town in England perhaps is so well supplied in this respect as Bedford is. Certainly, none is better supplied, and for this happy state of things the credit is largely due to the self-devotion and untiring energy of Mrs. Rawson, backed as it is by her staff of zealous and sympathetic trained nurses.

